# Preceding endoscopic submucosal dissection in submucosal invasive gastric cancer patients does not impact clinical outcomes

**DOI:** 10.1038/s41598-020-79696-y

**Published:** 2021-01-13

**Authors:** Kazutaka Kuroki, Shiro Oka, Shinji Tanaka, Naoki Yorita, Kosaku Hata, Takahiro Kotachi, Tomoyuki Boda, Koji Arihiro, Fumio Shimamoto, Kazuaki Chayama

**Affiliations:** 1 Department of Gastroenterology and Metabolism, Hiroshima University Hospital, Kasumi 1-2-3 Minami-ku, Hiroshima-shi, Hiroshima, Japan; 2 Department of Endoscopy, Hiroshima University Hospital, Hiroshima, Japan; 3 Department of Anatomical Pathology, Hiroshima University Hospital, Hiroshima, Japan; 4 Department of Faculty of Human Culture and Sciences, Hiroshima Shudo University, Hiroshima, Japan

**Keywords:** Gastrointestinal cancer, Gastric cancer, Gastrointestinal cancer

## Abstract

Submucosal deep invasion of gastric cancer (T1b2; depth of submucosal invasion ≥ 500 μm) is a risk factor for lymph node metastasis and, thus, is one of the criteria for curative treatment. Our aim was to evaluate the specific influence of endoscopic submucosal dissection (ESD) on the prognosis of patients with T1b2 gastric cancer. This was a retrospective analysis of 248 consecutive patients, with 252 pT1b2 gastric cancer lesions, who underwent ESD prior to additional surgery (Group A, n = 101) or surgery only (Group B, n = 147). After propensity score-matching (for sex, age, tumor diameter and gross type), we compared pathological characteristics between the 2 groups and the prognosis over a follow-up period ≥ 60 months. Compared to Group B, patients in Group A were older, with a higher proportion of men. The proportion of depressed and undifferentiated type tumors was greater in Group B than A, with larger tumor size and depth of submucosal invasion as well. There was no incidence of local recurrence, but distant metastasis was identified in 5% of cases in Group A and 3% in Group B. After propensity score-matching, there were no difference in the 5-year overall survival rate between Group A and B (87.5% vs. 91.2%, respectively), nor in the 5-year disease-specific survival rate (96.3% vs. 96.4%, respectively). ESD prior to surgery for T1b2 gastric cancer did not adversely affect clinical outcomes after additional surgery.

## Introduction

In Japan, gastric cancer is one of the most common cancers and the third most common cause of cancer-related death. Endoscopic submucosal dissection (ESD) was developed in the late 1990s and has been widely used for early gastric cancer (EGC) worldwide. ESD allows en bloc resection of even large EGCs and precise histologic assessment of the resected specimen, while being a less invasive treatment than surgical resection. The current Japanese Gastric Cancer Treatment Guidelines (version 5) for ESD were published by the Japanese Gastric Cancer Association (JGC) in 2018^1^. The new guidelines maintain the expanded criteria for ESD that were included in the previous guidelines: tumor size ≤ 30 mm; differentiated-type cancer; absence of vessel and lymphovascular involvement; and submucosal invasion < 500 μm. Additional surgery is also recommended in the new guidelines for gastric cancers with deep submucosal (SM) invasion (T1b2; depth of submucosal invasion ≥ 500 µm) identified in the pathological evaluation after ESD, due to the risk of lymph node (LN) metastasis.

The risk of LN metastases with gastric cancer with SM invasion < 500 µm (T1b1) ranges from 10.2–22.9%^2–7^, with the risk of LN metastases for gastric cancer with T1b2 invasion ranging between 10.6–26.8%^8–11^. The outcomes and prognosis of ESD for T1b1 gastric cancers have previously been reported^12,13^, with the usefulness and validity of ESD for T1b1 gastric cancers and EGC with ulceration having been demonstrated^14–16^. However, we identified only one study which examined the prognosis for patients with T1b2 gastric cancers who underwent ESD prior to additional surgery compared to those with T1b2 gastric cancers treated by surgical resection alone^17^. The evaluation of the usefulness and validity of ESD prior to additional surgery for T1b2 gastric cancers is currently limited by the significant differences in the clinical background of patients in the ESD and non-ESD groups in the study by Ojima et al.^17^, owing to wide range of indications for endoscopic treatment in the current guidelines. To address this limitation, we used propensity score-matching to evaluate the specific influence of ESD performed prior to additional surgery on the prognosis of patients with T1b2 gastric cancer.

## Methods

### Study group

**Figure. 1:**
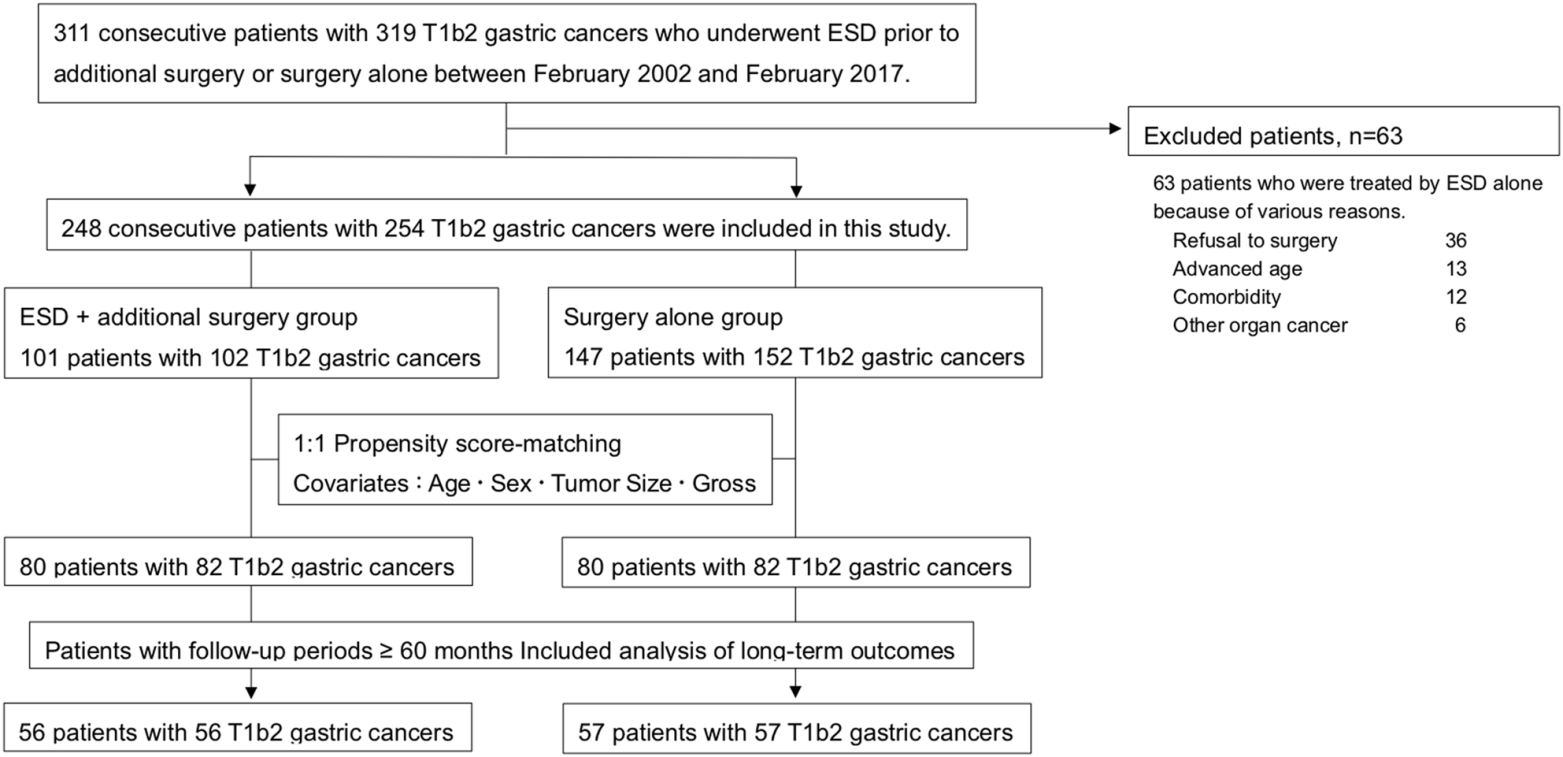
This figure showed the flow chart of patients and tumors included in this study. T1b2 gastric cancer; depth of submucosal invasion ≥ 500 µm, ESD; endoscopic submucosal dissection.

We retrospectively identified 311 consecutive patients with T1b2 gastric cancers who underwent ESD prior to additional surgery or surgery alone, between February 2002 and February 2017, at the Hiroshima University Hospital. Among patients who underwent ESD, those who did not meet the curative criteria of the JGC Guidelines were advised to undergo additional surgery. Of the 311 patients identified, 63 patients were treated using ESD only due to various reasons (refusal of surgery, comorbidity burden and/or advanced age) and were excluded. The remaining 248 patients (with 254 T1b2 gastric cancer lesions) were included in the analysis, 101 of whom underwent ESD prior to additional surgery (Group A) and the other 147 treated by surgical resection alone (Group B). In Group A, 78 patients (77%) enrolled in this study were diagnosed as M or SM1 cancer preoperatively, and the other patients were preoperatively diagnosed with SM2 cancer but they refused to undergo initial surgical resection due to old age, comorbidities, and/or activity of daily living status. According to the Japanese classification of gastric cancer, SM1 cancer was defined as a cancer which was preoperatively diagnosed as the depth of submucosal invasion < 500 µm, and SM2 cancer was defined as a cancer which was preoperatively diagnosed as the depth of submucosal invasion ≥ 500 µm. We first compared the clinicopathological features between Group A and B, and the clinical outcomes of ESD (operative time, en bloc resection, vertical margin, and complications) in Group A. En bloc resection was defined as resection in a single piece. Secondly, one-to one propensity score-matching was used to control for potential confounders for patients in Group A. After matching, 80 patients were identified in each group. Finally, we analyzed pathological characteristics between the 2 groups and the prognosis of patients over a follow-up period ≥ 60 months (Fig. 1).

Written informed consent was obtained from all patients prior to the treatment, and the study design was approved by the Ethics Committee of Hiroshima University Hospital. (No. E-1682).

The data collection and all experiments were performed in accordance with the relevant guidelines and regulations.

### ESD procedure

ESD was performed using a single-channel endoscope (H260 or H260Z, Q260J; Olympus Optical Co., Ltd., Tokyo, Japan, or EG-450RD5; Fujifilm Medical, Tokyo, Japan) or a two-channel scope (GIF-2TQ260M, Olympus, or EG-450D5; Fujifilm Medical). First, marking dots were placed on the normal mucosa, at approximately 5 mm from the tumor margin, to provide a safe margin. Second, after the local injection of a 10% glycerin solution and/or 4% sodium hyaluronate into the submucosa of the gastric wall, the mucosa around the lesion was incised circumferentially using an IT-knife or IT-knife2 (Olympus Medical Systems Co., Ltd., Tokyo, Japan). Third, dissection of the submucosal layer was performed using an IT-knife / IT-knife2 (Olympus Medical Systems Co., Ltd., Tokyo, Japan) or SB Knife-GX / SB Knife-Jr (Sumitomo Bakelite Co., Ltd., Tokyo, Japan). Finally, all vessels exposed to the ulceration after ESD were coagulated using hemostatic forceps (FD-410LR; Olympus Medical Systems Co., Ltd., Tokyo, Japan). Second-look endoscopy was consistently performed on the day after ESD. Once hemostasis of the vessels on the ulceration after ESD was confirmed, the patient was permitted to eat a light meal in the evening or the following day.

### Histopathological evaluation

The histopathological examination was based on the Japanese classification of gastric cancer. The specimens resected by ESD or those that were surgically resected were fixed with formalin and then sliced at 2-mm and 5-mm apart, respectively. The sections were stained with hematoxylin–eosin and then analyzed in detail. The histopathological type, tumor diameter, depth of submucosal invasion, lateral and vertical margins, and lymphovascular invasion were assessed for each slice. Immunohistochemical staining with antibodies against podoplanin (D2–40) was employed to distinguish small blood vessels from lymphoid capillaries to determine the presence of lymphatic invasion. Venous invasion was determined using Elastica van Gieson staining. The histopathological types were as follows: differentiated (well or moderately differentiated tubular adenocarcinoma or papillary adenocarcinoma) and undifferentiated (poorly differentiated tubular adenocarcinoma, mucinous adenocarcinoma, or signet ring cell carcinoma).

### Surveillance program after ESD and surgery

In both Group A and B, follow-up gastroduodenal endoscopy, laboratory measurements, including tumor markers, and chest and abdominal computed tomography were conducted annually after the index procedure. Recurrence was diagnosed based on imaging studies and histopathological findings.

### Measured variables

The following clinicopathological variables were evaluated for each group: sex, age, tumor location, tumor diameter, gross type, main histopathological type, and depth of SM invasion. These variables were compared between the two groups before and after propensity score-matching. The 5-year overall survival (OS) and 5-year disease-specific survival (DSS) rates for each group after propensity score-matching were assessed as long-term outcomes. The OS rate was defined as the percentage of patients who survived for a certain period after treatment. The DSS rate was defined as the percentage of patients who had not died of gastric cancer for a certain period after the index treatment.

### Statistical analysis

The chi-squared test and Fisher's exact tests were used to assess the association between various categorical variables and for intergroup comparisons of clinicopathologic characteristics. The survival period was defined as the period from the date of the index procedure (ESD or surgery) to the most recent date of confirming that the patient was alive or the date of the patient's death. The OS and DSS rates were estimated using the Kaplan–Meier method. Propensity scores were calculated using a logistic regression model with the variables of sex, age, tumor diameter, and gross type. After the propensity scores were estimated, one-to-one matching was performed using the nearest-neighbor method with a caliper set at 0.2. Further, *p* values < 0.05 was considered significant. All analyses were performed using JMP pro 14 software (SAS Institute, Cary, NC, USA).

## Results

### Clinicopathological features of T1b2 gastric cancers before propensity score-matching

The baseline characteristics for Group A and Group B, before propensity score-matching, are reported in Table 1, with significant differences summarized follows. Group A had a higher proportion of men than Group B (79% (80/101) vs. 63% (93/147), *p* < 0.01), and patients were also older in Group A than B (69.4 ± 10.1 years vs. 67.0 ± 11.5 years, respectively; *p* < 0.05). In Group A, 34% (34/101) of patients were over the age of 75 years, and 14% (14/101) over the age of 89 years. A higher proportion of patients in group B than A had tumors located in the middle third of the stomach (31% (32/102) vs. 57% (86/152); *p* < 0.01). Likewise, the proportion of depressed type tumors and undifferentiated type tumors was also greater in group B than A: depressed tumors, 65% (66/102) versus 78% (119/152), *p* < 0.05; and undifferentiated tumors, 21% (21/102) versus 49% (74/152), *p* < 0.01. The mean tumor diameter was larger in Group B than A (24.9 ± 18.0 mm (8-60 mm) vs. 32.6 ± 22.5 mm (7-150 mm), *p* < 0.01), as was the depth of SM invasion (1348 ± 841 µm (500–4250 µm) vs. 1855 ± 1087 µm (500–6000 µm), *p* < 0.01). The mean follow-up periods were 69.8 ± 39.8 months (1–174 month) in Group A and 75.7 ± 40.7 months (0–176 months) in Group B (*p* = 0.241). With regard to comorbidities in Group A, 8 patients (8%) had heart disease, 5 patients (5%) had cerebrovascular disease, and 8 patients (8%) had been treated for other types of cancer. LN metastasis was identified in 16 patients (16%) in Group A and 25 (16%) in Group B (*p* = 0.81).

**Table 1:** Clinicopathological features of T1b2 gastric cancer.

Variables	Total 248 cases 254 lesion	ESD + additional surgery 101 cases 102 lesion	Surgery 147 cases 152 lesion	P value
**Sex**				< 0.01
Male	173 (70)	80 (79)	93 (63)	
Female	75 (30)	21 (21)	54 (37)	
**Age (years)**	67.8 ± 11.0	69.4 ± 10.1	67.0 ± 11.5	0.048
**Location 1**				< 0.01
Upper third	62 (24)	30 (30)	32 (21)	
Middle third	118 (47)	32 (31)	86 (57)	
Lower third	68 (27)	37 (36)	31 (20)	
Remnant stomach	6 (2)	3 (3)	3 (2)	
**Location 2**				n.s
Anterior	50 (20)	17 (17)	33 (22)	
Posterior	61 (24)	21 (21)	40 (26)	
Greater curvature	63 (25)	27 (26)	36 (24)	
Lesser curvature	80 (31)	37 (36)	43 (28)	
**Tumor diameter (mm)**	29.5 ± 21.1	24.9 ± 18.0	32.6 ± 22.5	< 0.01
**Macroscopic type**				0.02
Elevated	69 (27)	36 (35)	33 (22)	
Depressed	185 (73)	66 (65)	119 (78)	
**Main histopathological type**				< 0.01
Differentiated	159 (63)	81 (79)	78 (51)	
Undifferentiated	95 (37)	21 (21)	74 (49)	
**Depth of SM invasion (μm)**	1651 ± 1024	1348 ± 841	1855 ± 1087	< 0.01
**Lymphatic invasion**				n.s
Positive	88 (35)	30 (29)	58 (38)	
Negative	166 (65)	72 (71)	94 (62)	
**Venous invasion**				n.s
Positive	42 (17)	16 (16)	26 (17)	
Negative	212 (73)	86 (84)	126 (83)	
**Lymph node metastasis**				n.s
Positive	41 (16)	16 (16)	25 (16)	
Negative	213 (84)	86 (84)	127 (84)	

### Clinical outcomes of ESD for T1b2 gastric cancers

The mean operative time was 105 ± 85 (range, 15–570) min. The en bloc resection rate was 91% (93/102), with 15% (15/102) of lesions having a positive vertical margin. In all cases, submucosal fibrosis was recognized in the resected specimen. Other characteristics of tumors in Group A were as follows (Table 2): 5% lesions (5/102) were located in the upper third of stomach and 1% (1/102) in the remnant stomach; and 4% (4/102) were undifferentiated type tumors. Bleeding occurred in 7% (7/102) lesions, with perforation in 5% (5/102). All cases could be treated conservatively, without additional surgery. Nine patients with 9 lesions in Group A (9%, 9/102) showed residual cancer in specimens resected by surgery after ESD and 16 patients with 16 lesions in Group A (16%, 16/102) showed lymph node metastasis in specimens resected by surgery after ESD.

**Table 2:** Clinical outcomes of ESD for T1b2 gastric cancer.

Variables	Total n = 102
Operation time (min)	105 ± 85
En bloc resection	93 (91)
Vertical margin positive	15 (15)
Bleeding after procedure	7 (7)
Perforation^a^	5 (5)

^a^All cases could be treated by conservative treatment.

### Prognosis after treatment, after propensity score-matching

**Figure. 2:**
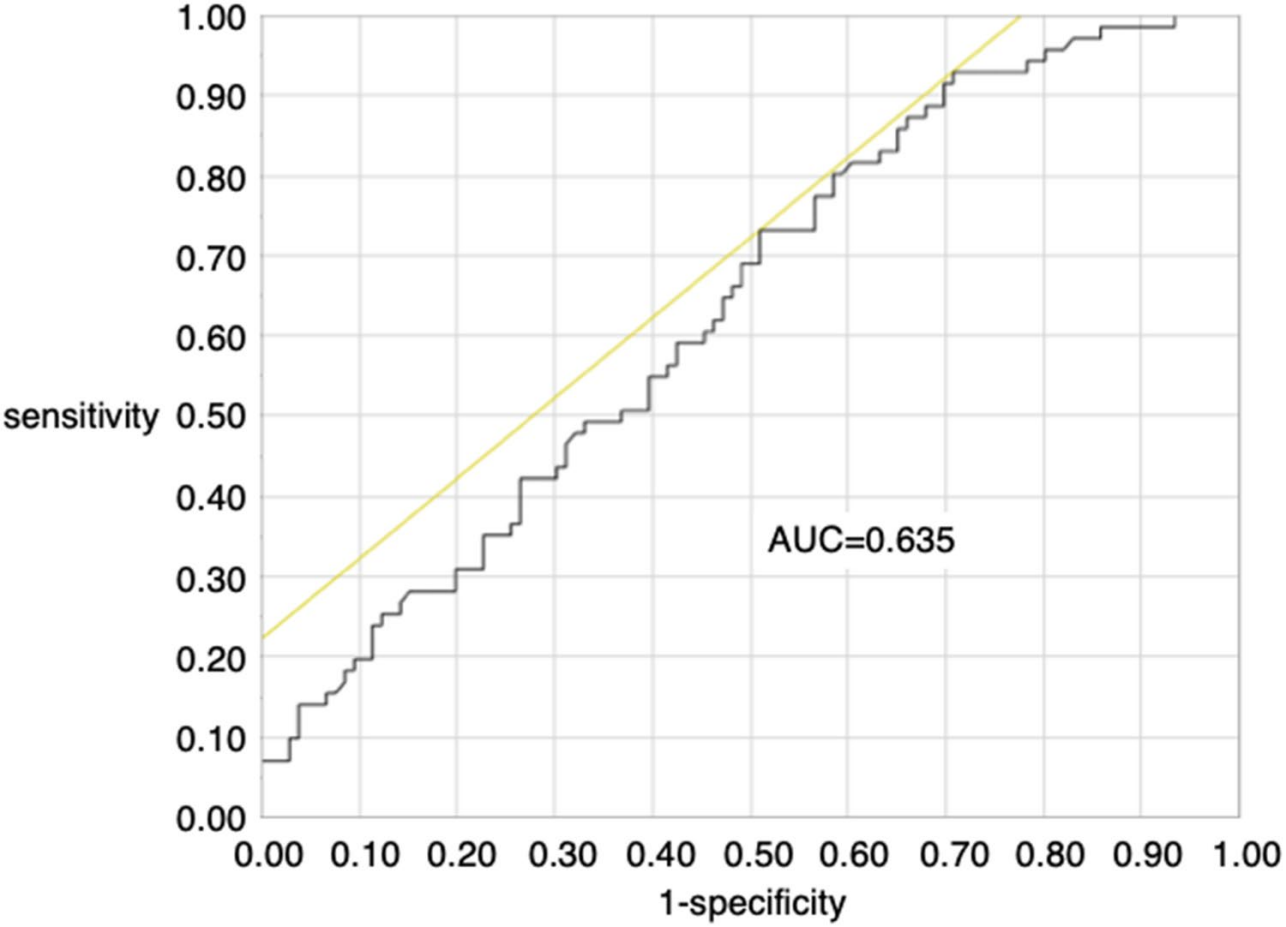
This figure showed the receiver operating characteristic curve for goodness of fit in propensity score model in this study. AUC; the area under the curve.

Figure 2 showed the receiver operating characteristic (ROC) curve for goodness of fit in this propensity score model. ROC curve was calculated by fitting a logistic regression model, using following clinically relevant variables; sex, age, tumor diameter, and gross type. The area under the curve was 0.635. After propensity score-matching, there was no significant difference in any of the variables between Group A and B (Table 3): sex, age, tumor location, tumor diameter, gross type, main histopathological type, depth of invasion. There was no local recurrence in either Group A or B. However, distant metastasis occurred in 5% (5/102) of cases in Group A and 3% (4/147) in Group B. The details of the cases with distant metastasis are reported in Table 4. Of note, among patients with distant metastasis, lymphovascular and/or vessel invasion was observed in 4 of the 5 patients in Group A and 3 of the 4 patients in Group B. All cases with distant metastasis within 5-years after treatment had the lymphatic vessel invasion and/ or vascular invasion. One of 5 patients with distant metastasis in Group A had no lymphovascular invasion, with distant metastasis observed at 79 months after the index treatment. The mean period to distant metastasis after treatment was 29 ± 29 (range, 7–98) months. Two of the patients with distant metastasis in Group A were alive at 98 and 27 months, respectively, with one of the patients with distant metastasis in Group B still alive at 71 months after treatment. The other patients with distant metastasis in Group A and B died of gastric cancer. Among the 3 patients with distant metastasis who were alive, LN metastasis was detected at 36 months after surgical resection in 1 patient, who subsequently underwent chemotherapy treatment, using Tegafur, Gimeracil and Oteracil Potassium, for a period of 34 months. The other 2 patients received supportive care. There was no significant difference in the rate of recurrence and the rate of death of gastric cancer between the two groups.

**Table 3:** Clinicopathological features of T1b2 gastric cancer after propensity score-matching.

Variables	Total 160 cases 164 lesion	ESD + additional surgery 80 cases 82 lesion	Surgery 80 cases 82 lesion	*p* value
**Sex**				n.s
Male	124 (78)	62 (78)	62 (78)	
Female	36 (22)	18 (22)	18 (22)	
**Age (years)**	68.9 ± 10.1	68.8 ± 10.2	69.0 ± 10.3	n.s
**Location 1**				n.s
Upper third	51 (31)	30 (37)	21 (26)	
Middle third	64 (39)	26 (32)	38 (46)	
Lower third	44 (27)	23 (28)	21 (26)	
Remnant stomach	5 (3)	3 (3)	2 (2)	
**Location 2**				n.s
Anterior	27 (16)	11 (14)	16 (19)	
Posterior	44 (27)	18 (22)	26 (32)	
Greater curvature	42 (26)	24 (29)	18 (22)	
Lesser curvature	51 (31)	29 (35)	22 (27)	
**Tumor diameter (mm)**	24.5 ± 12.3	24.6 ± 12.9	24.5 ± 11.8	n.s
**Macroscopic type**				n.s
Elevated	45 (27)	23 (28)	22 (27)	
Depressed	119 (73)	59 (72)	60 (73)	
**Main histopathological type**				n.s
Differentiated	113 (69)	63 (77)	52 (63)	
Undifferentiated	51 (31)	19 (23)	30 (37)	
**Depth of SM invasion (μm)**	1560 ± 936	1513 ± 750	1752 ± 1179	n.s
**Lymphatic invasion**				n.s
Positive	51 (31)	23 (28)	28 (34)	
Negative	113 (69)	59 (72)	54 (66)	
**Venous invasion**				n.s
Positive	27 (16)	12 (15)	15 (18)	
Negative	137 (84)	70 (85)	67 (82)	
**Lymph node metastasis**				n.s
Positive	25 (15)	13 (16)	12 (15)	
Negative	139 (85)	69 (84)	70 (85)

**Table 4:** Clinicopathological characteristics of 9 patients with recurrence after treatment for T1b2 gastric cancer.

Nos.	Sex	Age (years)	Tumor diameter (mm)	Gross type	Preoperative diagnosis	Treatment group	Main histopathological type	ly	v	SM invasion depth (μm)	Time to recurrence (month)	Recurrent organ	Prognosis
1	M	71	10	Elevated	M-SM1	A	Differentiated	+	+	1100	7	Liver	Death (17 M)
2	M	65	35	Elevated	SM2	A	Undifferentiated	+	+	4000	17	Bone	Death (32 M)
3	M	79	20	Elevated	M-SM1	A	Differentiated	–	–	1000	98	LN, Bone	Alive (98 M)*
4	M	69	40	Elevated	SM2	A	Differentiated	–	+	2000	7	Liver	Death (17 M)
5	M	77	45	Elevated	SM2	A	Differentiated	–	+	1300	14	LN	Alive (27 M)*
6	F	79	105	Depressed	SM2	B	Differentiated	+	+	2500	11	LN, liver	Death (24 M)
7	F	44	78	Elevated	SM2	B	Differentiated	+	–	3000	36	Peritoneum	Death (58 M)
8	M	61	10	Elevated	SM2	B	Differentiated	+	–	1500	36	LN	Alive (70 M)**
9	M	53	25	Depressed	SM2	B	Undifferentiated	–	–	1800	39	Peritoneum	Death (62 M)

M: male, F: Female, SM1: submucosal cancer with the depth of submucosal invasion < 500 µm, SM2: submucosal cancer with the depth of submucosal invasion ≥ 500 µm.

Group A: ESD + additional surgery, Group B: surgery, LN: lymph nodes, Death: Death of gastric cancer.

*Best Supportive Care **chemotherapy by TS-1.

**Figure. 3:**
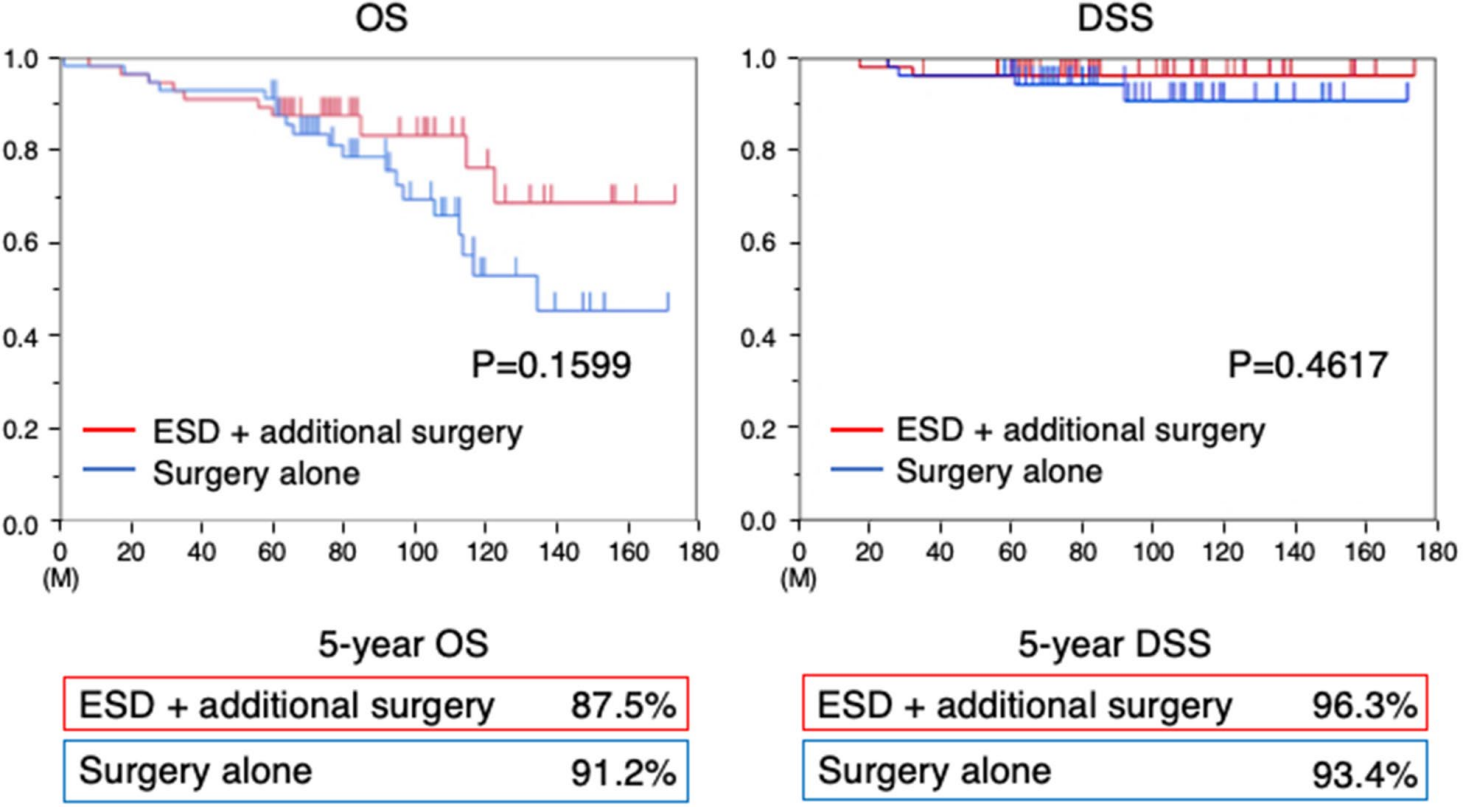
This figure showed Kaplan–Meier curves for the prognosis of the two groups. After propensity score-matching, there were no significant differences in 5-year OS rates and 5-year DSS rates after treatment of gastric cancer with submucosal deep invasion between two groups. OS; overall survival rate, DSS; disease specific survival rate.

After propensity score-matching, there were no difference between Group A and B in terms of the 5-year OS rate (87.5% vs. 91.2%, respectively,) and 5-year DSS rates (96.3% vs. 96.4%) after treatment of T1b2 gastric, over a mean follow-up of 87 ± 36 months (Figs. 3).

## Discussion

According to the JGC Association Treatment Guideline (version 5), there are several criteria for curative resection using ESD^1^, including the depth of invasion, tumor diameter, and ulceration. The combination of these criteria determines the conditions for curative resection because of the risk of LN metastasis. T1b2 gastric cancers, defined by a depth of mucosal invasion ≥ 500 µm alone, were excluded from the curative criteria for ESD, with additional surgery being recommended, with LN dissection, as LN metastasis negatively impacts the prognosis of patients with gastric cancer.

Previously, we reported on the outcomes and prognosis of ESD treatment for T1b1 gastric cancers (depth of SM invasion < 500 µm) and undifferentiated-type gastric cancers^12,13,18^. To the best of our knowledge, our study is the first to have evaluated the effect of preceding ESD prior to additional surgery on the prognosis of patients with T1b2 gastric cancer, after propensity score-matching.

In our study group, 101 patients, with 102 T1b2 gastric cancers, underwent proceeding ESD prior to additional surgery. There were 2 main reasons why we performed ESD prior to additional surgery in patients with T1b2 gastric cancer. The first is refusal of patients, and/or their family, to proceed with surgical resection as a first course of treatment due to age, comorbidities, and/or activity of daily living status^19^. It has previously been reported that 34–37% of patients with gastric cancer who were over the age of 80 years died of other comorbidities^20,21^. It has also been previously reported that the incidence of complication with ESD was not significantly difference between elderly and non-elderly patients^22^. Furthermore, compared to surgical resection, ESD facilitated preservation of the whole stomach, provided a better quality of life than did surgical resection, and provided a better quality of life for patients^23^. Based on this evidence, we did regard our decision to proceed with ESD resection of T1b2 gastric cancers as a feasible alternative to surgical resection in these cases, due to their advanced age and level of comorbidity. We performed conventional endoscopy, endoscopic ultrasonography (EUS), and chromoendoscopy for all gastric cancers, with the exception of obvious advanced cases of gastric cancer. A meta-analysis reported on the high sensitivity and specificity of accurate prediction of the depth of invasion of gastric cancer by EUS^24^. However, the pre-operative diagnosis of tumor invasion by conventional endoscopy and/or EUS does not have the same level of diagnostic performance, with a rate of accurate diagnosis of tumor invasion depth ranging between 62–81% for convention endoscopy and 67–85% for EUS^25–28^. Moreover, several studies have reported on the difficulty in performing accurate diagnosis for the lesions in the cardia of the stomach, as well as lesions with ulceration or perifocal inflammatory, and large lesions (> 30 mm)^24,29–31^. In our study, 78 lesions (77%) that were diagnosed as M or SM1 cancer were resect by ESD and finally diagnosed as T1b2 carcinoma by pathological examination.

Previous studies reported a rate of perforation during ESD procedure of 1.2–6.1%^32–35^. The risk factors for perforation include the location of the lesion in the stomach, tumor size, elevated macroscopic type, old age, submucosal fibrosis, and depth of invasion^35^. Of note, depth of invasion was reported as a risk factor for perforation for lesions with invasion of the muscularis mucosa. For lesions with submucosal invasion, the rate of perforations did not significantly differ between EGCs with mucosal invasion and those with submucosal invasion. In fact, in our study, intra-operative perforation occurred in 5 cases (5%, 5/102), with all 5 cases successfully treated using a conservative approach. Our results, therefore, indicate that ESD can be safely performed, even for T1b2 gastric cancer.

With regard to long term outcomes in Group A, the recurrence with distant metastasis occurred in 5 cases (5%, 5/102). Of these five cases with distant metastasis, 1 case had a positive vertical margin in ESD specimen (4%, 1/23) and 1 case had perforation during ESD (20%, 1/5).

LN metastasis is a risk factor for cancer recurrence. In our study, LN metastasis was identified in 16 patients (16%) in Group A and 25 (16%) in Group B (*p* = 0.81). This is consistent with the findings of previous studies that reported a rate of LN metastasis of 10–17% after surgical resection of T1b2 gastric cancer^36–40^. A depth of mucosal invasion ≥ 500 μm is, itself, a risk factor for LN metastasis. Several studies have reported the risk factors for LN metastasis to be a positive vertical margin, submucosal invasion, lymphovascular invasion, venous invasion, and undifferentiated-type cancers^12,40–43^. The 2018 JGC Treatment Guidelines adopted the eCura as risk scoring system for LN metastasis^1^. The eCura system included 5 clinicopathologic factors, with 3 points for lymphatic invasion and 1 point each for tumor size > 30 mm, positive vertical margin, venous invasion, and A depth of mucosal invasion ≥ 500 μm. A total score 0–1 is indicative of a low risk for LN metastasis, with a score of 2–4 indicative of an intermediate risk, and a score of 5–7 of a high risk. In Group A, 2 cases of LN metastasis were in the low risk group (4%: 2/49), with 4 cases being in the intermediate risk group (14%: 4/28), and 10 in the high-risk group (42%: 10/24). Finally, the overall survival rate and disease-specific survival rate after propensity score-matching was not significantly different between Group A and B. The results of this study suggest that the strategy of initial ESD may be acceptable prior to the additional surgical resection for SM2 gastric cancer preoperatively.

The limitations of our study need to be acknowledged. First, the results were obtained from a retrospective assessment based on the medical records of patients undergoing gastric ESD at a single cancer center in Japan. As such, a selection bias cannot be denied. We did perform a propensity score-matching analysis to minimize differences between the two groups. Second, the sample size after propensity score-matching was relatively small as the study was conducted in a single center and there were large differences in the background characteristics between the two groups. Thus, a prospective multicenter study over a period of 5 years is required to more precisely evaluate the clinical outcomes of ESD in patients with T1b2 gastric cancer. Third, the cut intervals of the specimens resected by ESD and surgery are different (2 mm vs. 5 mm). This may affect the histopathological examination. Fourth, in this study, many lesions enrolled in Group A were diagnosed as M or SM1 cancer preoperatively. Ideally, the inclusion in group A is considered to be limited to the patients with SM2 who rejected initial standard gastrectomy.

In conclusion, ESD for patients with T1b2 gastric cancer does not adversely impact clinical outcomes after additional surgery, so long as an appropriate vertical margin can be obtained during resection.

## Abbreviations

ESD: Endoscopic submucosal dissection
EGC: Early gastric cancer
JGC: Japanese Gastric Cancer Association
SM: Submucosal
LN: Lymph node
OS: Overall survival
DSS: Disease-specific survival
ROC curve: Receiver operating characteristic curve
